# Persistence of *Plasmodium falciparum* DNA detection after antimalarial treatment and the implication of gametocytes: a prospective cohort study in a non-endemic setting

**DOI:** 10.1128/spectrum.02312-26

**Published:** 2026-09-02

**Authors:** Azza Bouzayene, Valentin Joste, Tristan Candau, Rizwana Zaffaroullah, Véronique Sarrasin, Nicolas Argy, Sandrine Cojean, Sandrine Houzé

**Affiliations:** 1 Université Paris Cité, IRD, Inserm, MERIT 555089, Paris, France; 2 Centre National de Référence du Paludisme, AP-HP, Hôpital Bichat Claude Bernard 55076 , Paris, France; 3 Service de parasitologie et mycologie, AP-HP-, Hôpital Bichat Claude Bernard 55076 , Paris, France; 4 Équipe Rate, Infections et Parasites, Institut Pasteur 27058, Paris, France; 5 Université Paris-Saclay 27048, Gif-sur-Yvette, France; 6 UMR BIPAR, Animal Health Laboratory, ANSES, INRAE, École Nationale Vétérinaire d'Alfort, Université Paris-Est 55036 , Maisons-Alfort, France; University of Kentucky, Lexington, Kentucky, USA

**Keywords:** malaria, *Plasmodium falciparum*, molecular diagnosis, therapeutic efficacy, PCR positivity persistence, gametocytes, non-endemic setting

## Abstract

Highly sensitive molecular assays are increasingly used for malaria diagnosis but are not recommended for follow-up, as *Plasmodium falciparum* DNA detection may persist after effective antimalarial treatment, complicating these tests’ clinical interpretation. The biological basis of this persistence remains poorly defined. We investigated the potential role of gametocytes in the context of treated infections. In a prospective cohort of 100 successfully treated patients, we analyzed 379 blood samples collected up to day 28 (D28) post-treatment. We used quantitative PCR (qPCR) to detect parasite DNA targeting *PfvarATS*, a mainly asexual-stage marker, and *18S ribosomal RNA* (*18S rRNA*), a pan-stage marker. To differentiate residual nucleic acid detection from ongoing parasite transcriptional activity among patients’ samples that remained DNA-positive at D28, we used RT-qPCR targeting *PfvarATS*, *18S rRNA*, and gametocyte-specific transcripts (*PfAP2-G*, *Pfs16*, *PfMGET*, and *PfCCP4*). After anti-malarial treatment, 61% of samples remained qPCR positive at D28. *PfvarATS* transcripts declined sharply (12% positivity rate at D28), while *18S rRNA* transcripts persisted longer (60% at D28). Gametocyte-specific markers were detected in a subset: *Pfs16* (immature stages) was frequent early (100% at D0 and 94% at D3), while mature gametocyte markers (*PfCCP4/PfMGET*) peaked at D7 (33%) and were still detectable in 12% of samples at D28. Therefore, most D28 PCR positivity (88%) occurred without detectable mature gametocyte transcripts. Persistent *P. falciparum* DNA detection post-treatment in a non-endemic setting predominantly reflects non-viable parasites or residual genetic material and a minority of circulating mature gametocytes. This highlights a key diagnostic pitfall: post-treatment PCR positivity does not equate to treatment failure. Results must be interpreted with clinical context, timing, and assay targets.

**IMPORTANCE:**

This study confirmed that persistent *P. falciparum* DNA detection after effective antimalarial treatment mainly reflects residual parasite material, challenging the use of PCR in treatment efficacy follow-up. By excluding mature gametocytes as the primary source of persistent DNA detection, this study advances our understanding of post-treatment parasite nucleic acid kinetics in the organism and supports the importance of investigating alternative biological mechanisms.

## INTRODUCTION

Malaria remains a major global health burden, with an estimated 282 million cases and 610,000 deaths reported in 2024, mainly in the African region (1). In metropolitan France, based on the number of malaria cases reported by the French National Malaria Reference Center, it is estimated that nearly 6,000 cases of malaria were diagnosed in 2024, the majority occurring in travelers returning from sub-Saharan Africa (2).

This disease is caused by mosquito-transmitted protozoan parasites of the genus *Plasmodium*. Six main species infect humans, including *Plasmodium falciparum*, *Plasmodium vivax*, *Plasmodium malariae*, *Plasmodium ovale wallikeri*, *Plasmodium ovale curtisi*, and *Plasmodium knowlesi*. The greatest clinical burden worldwide is attributable to *P. falciparum* and *P. vivax* (3). Effective management of malaria includes prompt diagnosis and treatment, but controlling the disease remains challenging due to emerging drug resistance, diagnostic difficulties, and the biological complexity of the parasite’s life cycle.

A critical stage in the *Plasmodium* life cycle is the development of gametocytes, the sexual forms of the parasite that are transmissible to mosquitoes, thereby sustaining the disease’s transmission (4). Contrary to other *Plasmodium* species, gametocytogenesis is delayed for *P. falciparum*, and it involves several stages, from early immature (stages I–IV) to mature forms (stage V). Immature gametocytes are sequestered in deep tissues such as the bone marrow, and once mature, they are released into the bloodstream 7–10 days post-infection and are capable of infecting mosquitoes (4). Morphologically, the early immature stages of gametocytes are indistinguishable from the young trophozoite stages; however, several molecular markers of early-stage gametocytes have been identified, including *Pfs16*, *Pfg27*, and *PfGEXP-5* (5). Additionally, a master regulator of sexual commitment has been well characterized in *P. falciparum* and *Plasmodium berghei* species, *PfAP2-G* of the ApiAP2 DNA-binding protein family (6).

The molecular diagnosis of malaria has revolutionized its detection, allowing the identification of plasmodial DNA with high sensitivity through nucleic acid amplification (NAT) techniques like polymerase chain reaction (PCR) and loop-mediated isothermal amplification (LAMP) (7). These techniques have proven useful not only for diagnosing symptomatic infections but also for identifying submicroscopic and asymptomatic carriers, who serve as silent reservoirs of transmission (8). However, these methods cannot distinguish between DNA originating from viable parasites and residual DNA from non-viable parasites. As a result, residual genetic material from dead parasites persisting in the bloodstream after treatment can lead to “false” positive test results (9). This issue complicates diagnostic interpretation, assessment of treatment efficacy, and surveillance of prevalence and transmission. According to the World Health Organization guidelines, monitoring the therapeutic efficacy of malaria treatment focuses on the evaluation of clinical and parasitological responses for at least 28 days post-treatment (at days 3, 7, and 28), mainly by confirming the absence of detectable parasitemia in the blood by microscopy (10). This reflects the widespread availability, affordability, and practicality of microscopy in routine clinical practice and endemic settings.

While current antimalarial treatments, such as artemisinin-based combination therapies (ACTs), are effective against the asexual blood stages of the parasite, mature gametocytes can persist despite treatment, contributing to ongoing transmission. In a meta-analysis pooling data from 121 clinical trials, 1.9% of patients without initial gametocytes developed them by day (D) 28 post-ACT (11). Moreover, the proportion of gametocyte appearance varied by ACT regimen; risk was lowest with artemether-lumefantrine (AL) and artesunate-mefloquine, but higher with dihydroartemisinin-piperaquine (DP) and artesunate-amodiaquine treatments (11). In addition, non-ACTs (sulfadoxine-pyrimethamine, chloroquine, or quinine [Q]) have been associated with a delayed but more pronounced gametocytogenesis (12–14), while atovaquone-proguanil (AP)-based treatment has demonstrated sporontocidal activity and thus transmission-blocking potential (15). To this day, only primaquine and methylene blue have approved gametocytocidal activity, but side effects limit their use (16, 17).

Related research has demonstrated that quantitative PCR (qPCR) analysis during follow-up shows slow clearance of *P. falciparum* DNA in one-third of Kenyan children treated with ACT (18) and highlighted the importance of addressing residual gametocyte carriage in treated individuals to reduce the risk of transmission. Another study conducted in a nonendemic setting investigated the utility of NAT techniques in monitoring the efficacy of antimalarial treatment through patient follow-up (19). The results showed positive detection of parasite DNA in 12% and 10% of samples at D28 with the nested conventional PCR and the qPCR coupled with the high-resolution melting method, respectively. These findings highlighted the higher sensitivity of molecular methods compared to microscopy in the context of treatment efficacy evaluation and the importance of careful PCR results interpretation, notably in nonendemic settings. Alternative explanations for residual DNA detection were also suggested, mainly the implication of sexual parasite stages (19, 20).

Thus, it is important to differentiate gametocytes and other parasite stages and to distinguish residual non-viable genetic material from viable parasites. Since asexual and sexual blood stage parasites possess identical genomic DNA (gDNA), DNA-based assays cannot differentiate them. Accordingly, discrimination relies on stage-specific transcriptional activity, notably messenger RNA (mRNA) expression. Furthermore, unlike genomic DNA, which may persist after parasite death, mRNA is continuously synthesized and degraded through tightly regulated RNA turnover pathways in eukaryotes (21). Consequently, detection of stage-specific mRNA transcripts is generally considered a stronger indicator of metabolically active parasites rather than DNA detection alone, although transcript stability varies between genes and should not be regarded as an absolute measure of viability.

In this study, we investigated the role of *P. falciparum* gametocytes in the prolonged NAT positivity in patients in France with travel-related imported malaria. To test this, we prospectively monitored treated patients using a multi-marker molecular approach targeting both asexual and sexual (early and mature) stages to understand the origins of persistent DNA detection.

## MATERIALS AND METHODS

### Clinical samples

This longitudinal prospective monocentric study included EDTA-collected whole blood samples from patients monitored at Bichat Claude Bernard Hospital (Paris, France) between March 2020 and April 2023. Diagnosis was made by microscopy and confirmed by species qPCR as monospecific *P. falciparum* malaria on day 0 samples (prior to treatment). After treatment, patients were followed and sampled at predefined time points, at approximately days 3, 7, and 28 after treatment initiation. Throughout the manuscript, the time points are referred to as D0, D3, D7, and D28, with the numbers in parentheses indicating the actual collection dates for each visit. Patients were included in this study if at least D0 and/or D3 and D28 blood samples were available, as some patients did not attend all scheduled intermediate visits.

Upon receipt, follow-up samples were subjected to routine biological analysis, including microscopy on Giemsa-stained thin and thick blood smears, as well as LAMP and DNA and RNA extractions. Whole blood samples were stored at 4°C, while microscopy and LAMP analyses were performed and subsequently processed for DNA and RNA extractions. Blood intended for DNA extraction was maintained at 4°C until processing, whereas aliquots prepared in TRIzol for RNA extraction were stored at −80°C until extraction.

**Fig 1:**
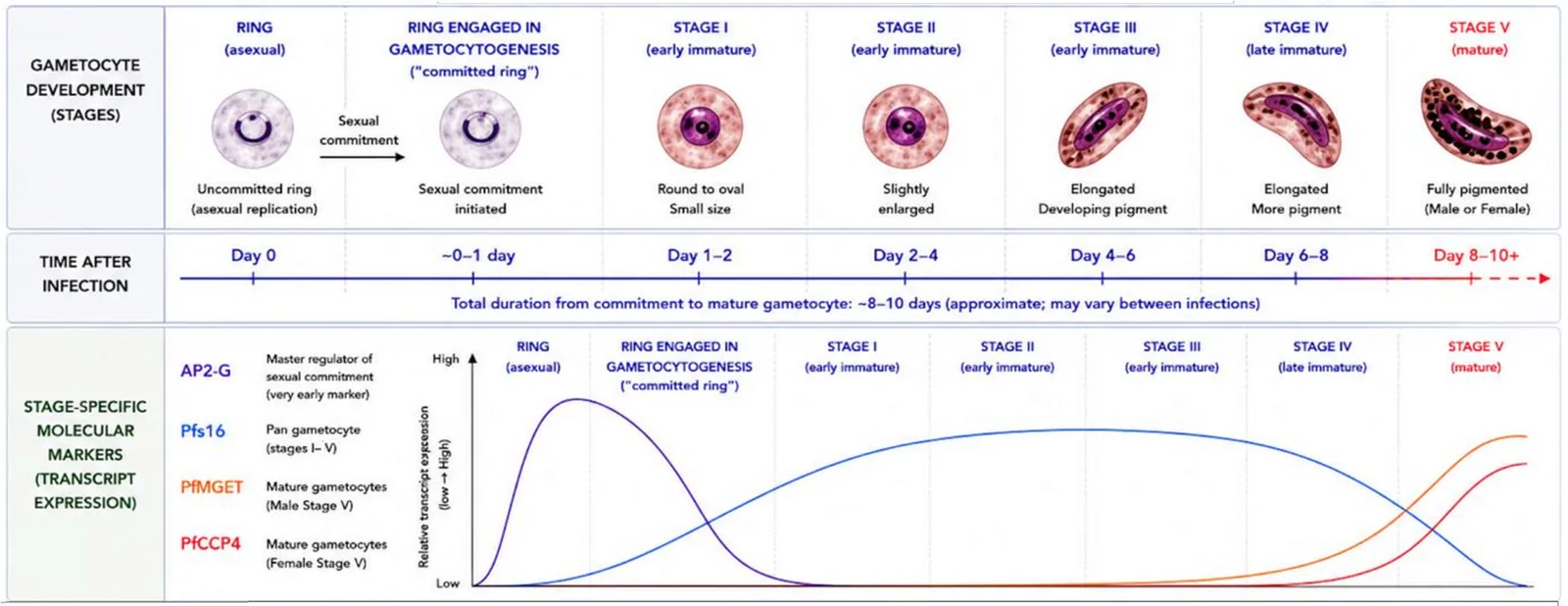
Literature-based schematic representation of *Plasmodium falciparum* gametocyte development, the temporal expression windows of the molecular markers used in this study, and the rationale underlying the RT-qPCR assays. The developmental stages and marker expression windows are schematic and derived from published literature (4, 6, 23–25); they are intended to illustrate the biological rationale for marker selection and are not quantitative representations of transcript abundance.

DNA extracts were analyzed using *PfvarATS/18S rRNA* multiplex qPCR assay. RNA was transcribed and analyzed with the PfvarATS/18S rRNA multiplex qPCR, the mature gametocyte markers *PfCCP4/PfMGET* multiplex RT-qPCR, and the early gametocyte markers *PfAP2-G* and *Pfs16* RT-qPCRs. *PfCCP4* and *PfMGET* were evaluated throughout follow-up (D0–D28) to assess the presence of mature gametocytes. In contrast, *PfAP2-G* and *Pfs16* were evaluated during the early follow-up period (D0–D7), as these markers are associated with sexual commitment and early gametocyte development. *PfAP2-G*, the master regulator of sexual commitment, is transiently expressed in sexually committed rings (6), and thus, its expression was anticipated to be largely restricted to D0 samples and, as a stretch, on D3 samples. *Pfs16* expression was assessed during the early follow-up period (D3–D7) because it is an early gametocyte marker expressed from stage I onward, with predominant expression during immature gametocyte development (22). By comparing it with the mature gametocyte markers *PfCCP4* and *PfMGET*, we aimed to distinguish the presence of immature versus mature gametocytes during post-treatment follow-up. To clarify the biological rationale underlying our experimental design, Fig. 1 summarizes the timing of gametocyte development and the temporal expression windows of the molecular markers used in this study.

The presented RNA analysis results concern only patients whose D28 samples were qPCR-*PfvarATS* DNA positive.

All clinical and epidemiological data were collected from the French National Malaria Reference Center’s database and analyzed in accordance with the common public health mission of all the National Reference Centers of France. Everything was coordinated with the “Santé Publique France” organization for malaria surveillance and care. According to article L1221-1.1 of the public health code in France, the study of biological samples obtained from routine medical care is considered non-interventional research and therefore only requires the non-opposition of the patient during sampling (per article L1211-2 of the public health code). The data collected were anonymized before use.

### LAMP method

The commercial LAMP-based assay Alethia Malaria (Launch Diagnostics, France) is a qualitative isothermal molecular test allowing the direct detection of *Plasmodium spp*., without species identification, by targeting segments of its mitochondrial DNA after lysis of whole blood samples. Analytical sensitivity assays performed by the manufacturer showed a limit of detection (LoD) of 2 parasites/μL for *P. falciparum*. This test was performed on the Illumipro-10 (Launch Diagnostics, France) automated isothermal amplification and detection system following the test procedure provided by the manufacturer. It was systematically conducted on all samples upon receipt to determine the presence or absence of plasmodial DNA.

### DNA extraction

For all included samples, DNA was extracted from 200 μL of whole blood and eluted in 100 μL of buffer using the EMAG (bioMérieux, France) extractor following a total nucleic acids extraction from whole blood protocol adapted from the manufacturer. DNA was then stored at −20°C until analysis.

### RNA extraction and reverse transcription-PCR

For all included samples, RNA extraction was performed on red blood cell pellet to enrich erythrocytes and minimize contamination by abundant leukocyte RNA. This approach was chosen to improve the detection of *Plasmodium* transcripts. A total of 200 μL of red blood cell pellet was resuspended in five times the volume of TRIzol Reagent (Thermo Fisher Scientific, USA). RNA was then extracted by the addition of 200 μL chloroform (Sigma-Aldrich, USA) followed by centrifugation at 12,000 *g* for 15 min at 4°C. The aqueous layer containing RNA was purified using the Pure Link RNA Mini Kit (Thermo Fisher Scientific, USA) following the manufacturer’s instructions with an additional on-column DNA removal step using the Pure Link DNase solution (Thermo Fisher Scientific, USA). Before reverse transcription-PCR (RT-PCR), RNA samples were analyzed by qPCR to rule out contamination with genomic DNA before being stored at −80°C. All samples were confirmed negative before use.

Complementary DNA (cDNA) was generated with the SuperScript II reverse transcriptase kit (Thermo Fisher Scientific, USA) by using oligo(dT)_12–18_ (Thermo Fisher Scientific, USA) following the procedure provided by the manufacturer.

### qPCR *PfvarATS/18S rRNA*

All DNA and cDNA samples were analyzed using an in-house qPCR multiplex assay adapted from Schindler et al. as previously described (7, 26). Each reaction was performed in a final volume of 20 µL, including 6 µL of DNA or cDNA template. This assay targets two independent *Plasmodium* genes: the Pan-*Plasmodium 18S rRNA* sequence and the *P. falciparum*-specific acidic terminal sequence of the *var* genes (*PfvarATS*). The *HsRNaseP* human gene was used as an internal control to rule out extraction failure. Oligo sequences and reaction details are provided in Tables S1 and S2.

### RT-qPCR *PfCCP4/PfMGET*

To determine the presence of *P. falciparum* gametocytes in follow-up samples positive by the *PfvarATS* target, cDNA samples were analyzed using a multiplex RT-qPCR assay targeting the female and male gametocyte genes *PfCCP4* and *PfMGET*, respectively, expressed by mature stage V gametocytes as described by Meerstein-Kessel et al. (25). This assay was performed in a final volume of 20 µL, including 5 µL of cDNA template. Oligo sequences and reaction details are provided in Tables S1 and S2. Although no internal control was included in this assay, the cDNA extracts were validated by amplification of the human *HsRNaseP* gene in the *PfvarATS/18S rRNA* qPCR assay. In addition, each run included positive and negative controls to validate assay performance and exclude contamination.

### RT-qPCR *PfAP2-G/Pfs16*

To assess sexual conversion in our samples, we developed a TaqMan qPCR assay targeting the AP2 domain transcription factor gene (*AP2-G*) (PF3D7_1222600) and the sexual stage-specific protein precursor gene (*Pfs16*) (PfNF54-040011900) expressed by sexually committed rings and immature gametocytes, respectively. *PfAP2-G* expression was assessed on available D0 and D3 samples, whereas *Pfs16* was analyzed on available D0, D3, and D7 samples for which RNA quantity was sufficient. The gene sequences were obtained from PlasmoDB, and the corresponding probes and primers were designed using the Primer3 program. Oligo sequences and reaction details are provided in Tables S1 and S2. No internal control was also included in these assays.

### Specificity and sensitivity analysis

The specificity of the *PfAP2-G* and *Pfs16* primers and probes was assessed *in silico* using BLAST searches (http://blast.ncbi.nlm.nih.gov) to verify *P. falciparum* specificity. Experimentally, we tested malaria-negative human blood samples (*n* = 5) and DNA of *P. ovale* (*n* = 3), *P. malariae* (*n* = 3), *P. vivax* (*n* = 3), and *P. knowlesi* (*n* = 1). For the *PfCCP4/PfMGET* RT-qPCR, we used asexual stages of the NF54 culture strain, a clinical strain originally isolated from a Dutch woman who contracted malaria in Tanzania in the late 1970s (27) and is the reference strain used for *P. falciparum* gametocyte studies.

The sensitivity of RT-qPCR for transcript detection was evaluated using serial dilutions of *in vitro* cultured asexual and sexual *P. falciparum* stages (NF54 strain). Early and mature gametocytes were obtained from a culture of the NF54 strain induced to undergo gametocytogenesis as previously described (28) and quantified per microliter by microscopic counting using a Malassez counting chamber. Gametocyte counts were converted to gametocytes per microliter, and RNA was subsequently extracted from these preparations, reverse-transcribed into cDNA, and serially diluted 10-fold to determine the LoD of each marker. These samples were assessed in triplicate, given the limited amount of cDNA available to test the multiple assays. Although an independent molecular assay specifically confirming the complete absence of asexual stages was not performed, gametocyte culture was generated using the standard N-acetylglucosamine (NAG) treatment protocol. This treatment selectively eliminates proliferating asexual blood stages by blocking erythrocyte invasion while allowing committed stages to continue their maturation. Following NAG treatment, Giemsa-stained smears showed only gametocyte stages and no asexual parasites.

### Statistical analysis

Graphical representations and statistical analyses were performed using GraphPad Prism v10.6.1 (GraphPad Software, San Diego, CA, USA). For each follow-up time point, the proportion of positive samples was calculated and expressed as a percentage with 95% confidence intervals, computed using the Wilson method for binomial proportions. Binary outcomes (positive/negative) were used to calculate the proportion of positive samples at each follow-up time point, with corresponding 95% confidence intervals.

Data were analyzed as pooled cross-sectional data sets rather than paired longitudinal observations. Comparisons of positivity rates between the baseline time point (D0) and subsequent follow-up days were performed using Fisher’s exact test. A two-sided *P* value < 0.05 was considered statistically significant.

## RESULTS

### RT-qPCR analytical sensitivity

All samples analyzed for the specificity test were negative. We tested cDNA of asexual parasite cultures with the *PfCCP4/PfMGET* RT-qPCR and, as already reported by Meerstein-Kessel et al., when asexual parasite densities exceed 1,000 parasites/µL, low-level gametocyte transcripts could be detected (25). This was not considered a limitation for our study, as our cohort consisted of treated follow-up samples that were microscopy negative and thus did not contain viable high-density asexual forms. This was also observed with the *Pfs16* RT-qPCR assay at D0 samples, likely reflecting the presence of a proportion of sexually committed parasites from previous cycles expressing early gametocyte transcripts. To exclude any minor contribution from residual genomic DNA following DNase treatment, RNA extracts were controlled by qPCR before reverse transcription.

RT-qPCR sensitivity assays demonstrated an LoD of 0.4 parasites/µL for *PfAP2-G*, 0.04 parasites/µL for *Pfs16*, 1.5 gametocytes/µL for *PfCCP4/PfMGET*, 0.06 gametocytes/µL for *18S rRNA*, and 0.6 gametocytes/µL for the *PfvarATS*.

Previous assays on asexual forms demonstrated an LoD of 0.05 parasites/µL for *PfvarATS* and 0.5 parasites/µL for *18S rRNA* (26).

### Patient cohort and study design

A total of 100 adult patients were included between 2020 and 2023 (60 males and 40 females), but only 65 had a complete follow-up (at least D0/D3 and D28). All patients returned from sub-Saharan African countries. Baseline parasitemia at D0, as determined by microscopy, ranged from 0 (positive by LAMP and PCR) to 1.2 million parasites/µL. Treatments were ACTs (*n* = 91; 72 DP, 15 AS then DP, and 4 AL), AP (*n* = 5), or quinine (*n* = 4). No therapeutic failures occurred. Clinical, epidemiological, and parasitological data are presented in supplementary data.

**Fig 2:**
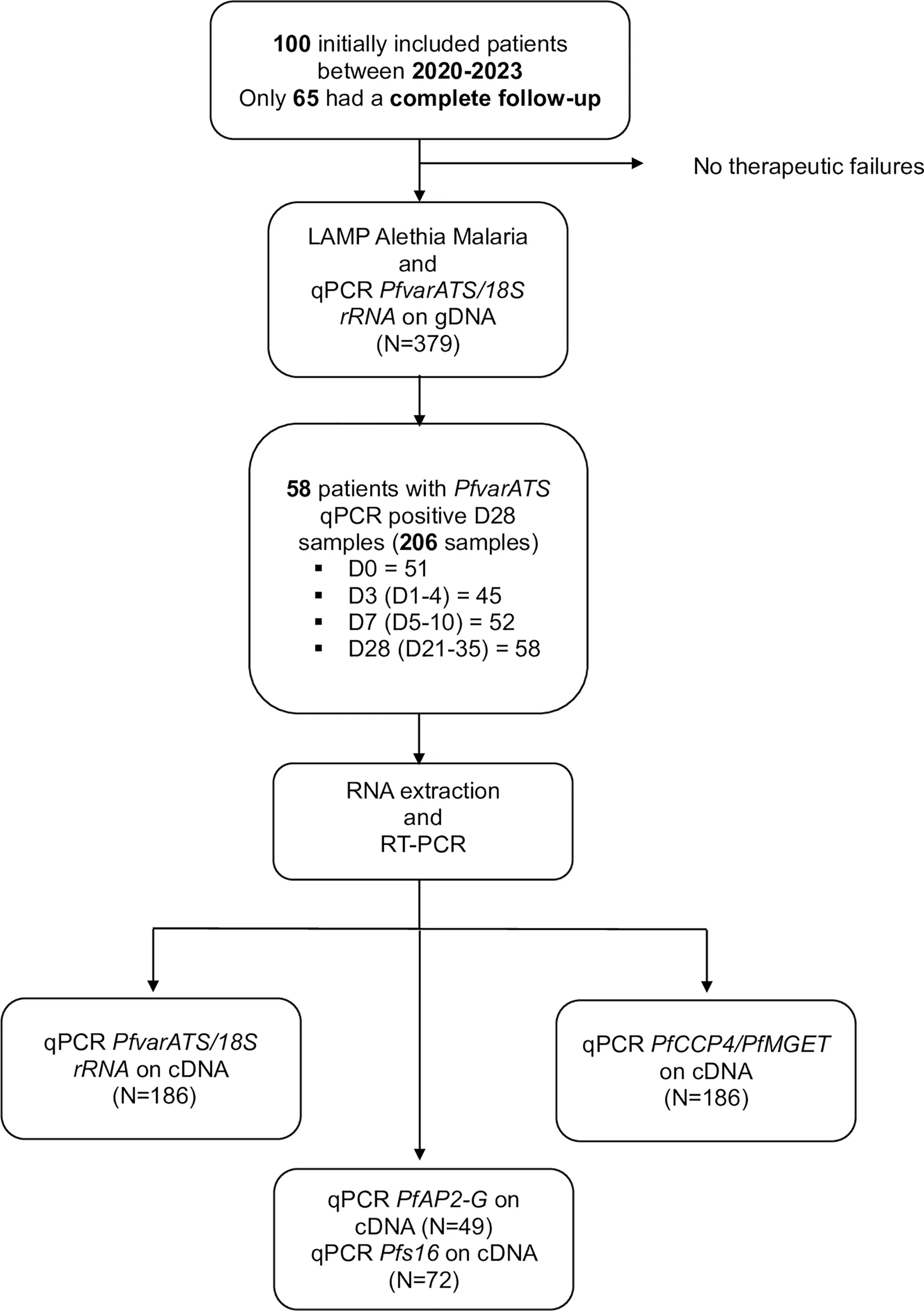
Study design and sample processing workflow. Among the 100 patients initially enrolled between 2020 and 2023, only 65 completed follow-up. No therapeutic failures were observed. A total of 379 follow-up blood samples were screened using the Alethia Malaria LAMP assay and qPCR targeting *PfvarATS* and *18S rRNA*. RNA extraction and RT-PCR analyses were performed on patients who had *PfvarATS* qPCR-positive samples at D28 (*n* = 58), corresponding to 206 follow-up samples collected at D0 (*n* = 51), D3 (*n* = 45), D7 (*n* = 52), and D28 (*n* = 58). cDNA was successfully generated for 186 samples and analyzed using *PfvarATS*/*18S rRNA* qPCR and *PfCCP4/PfMGET* qPCR. In addition, *PfAP2-G* and *Pfs16* assays were performed on a subset of the cohort, depending on template availability, at D0, D3, and D7.

All samples were first screened by the LAMP method to determine the presence or absence of plasmodial DNA. In parallel, the *PfvarATS/18S rRNA* multiplex qPCR was performed on DNA extracts to confirm the presence of *P. falciparum* genomic DNA (Fig. 2). Following this screening, we identified 58 patients (206 samples) who had a positive follow-up by qPCR up to D28 post-treatment. RNA was extracted from these follow-up samples. Reverse transcription failed for 20 samples; therefore, only 186 cDNA samples were ultimately analyzed by the *PfvarATS/18S rRNA* and *PfCCP4/PfMGET* RT-qPCR assays. Early follow-up samples (D0, D3, or D7) were screened for early gametocyte markers (*PfAP2-G/Pfs16*). However, because these assays were implemented later in the study, sufficient RNA material was not available for all participants. Consequently, *PfAP2-G* and *Pfs16* RT-qPCR analyses were conducted on subsets of the initially selected cohort: *PfAP2-G* on 35 of 51 D0 and 14 of 45 D3 samples, and *Pfs16* on 10 of 51 D0, 16 of 45 D3, and 46 of 52 D7 samples (Fig. 2).

### Persistence of *Plasmodium* DNA post-treatment

**Fig 3:**
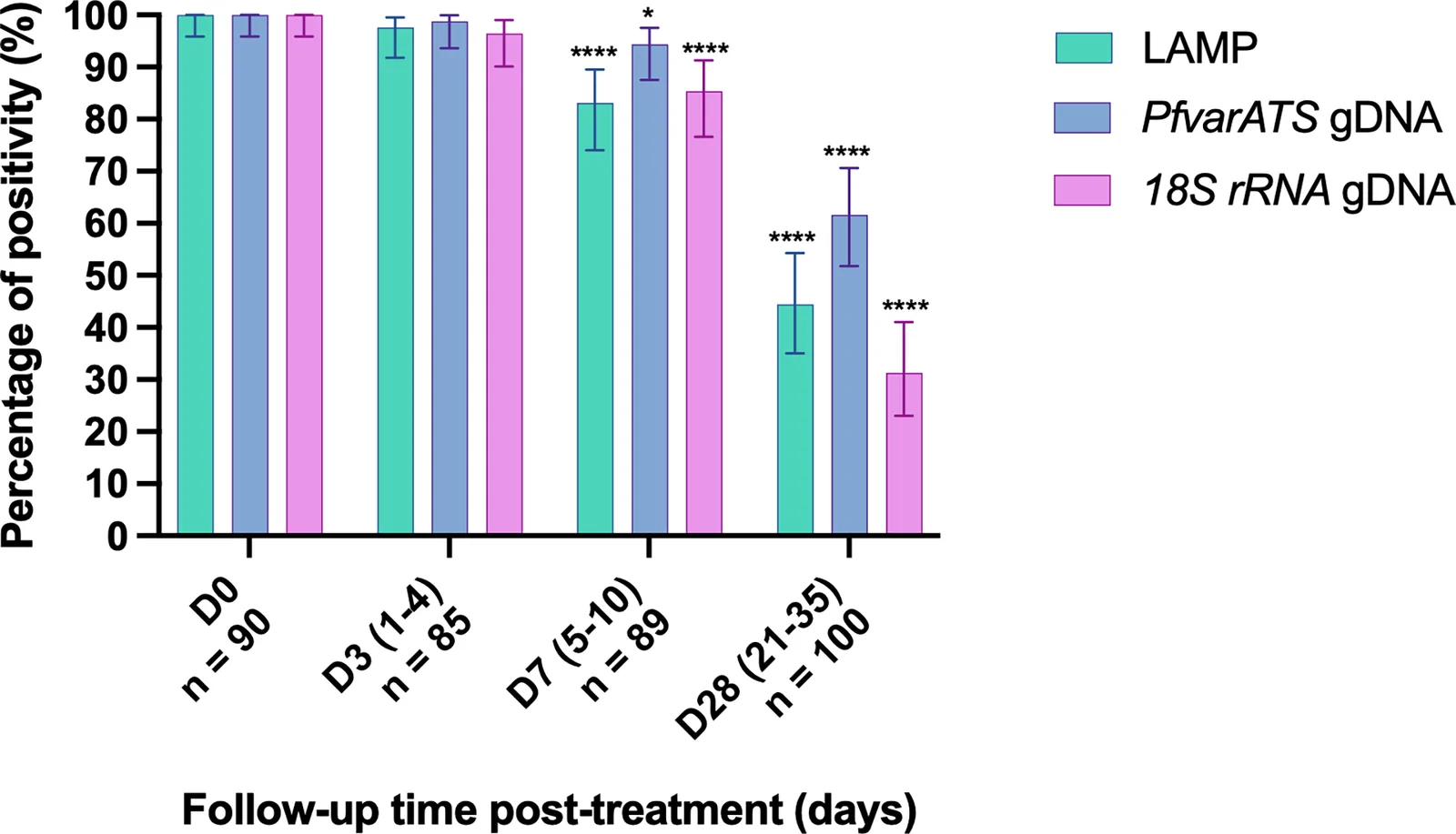
Plasmodium DNA detection during follow-up by LAMP and PfvarATS/18S rRNA qPCR. Included samples were analyzed using the LAMP assay and qPCR at D0 and during post-treatment follow-up up to D28. At each follow-up time point, samples were classified as positive or negative, and the proportion of positive samples was calculated relative to the total number of samples tested at that time point. Results are expressed as percentages with 95% confidence intervals (upper and lower bounds) and were analyzed using GraphPad Prism v10.6.1. Statistical comparisons between follow-up time points and baseline (D0) were performed using Fisher’s exact test, *P =* 0.03 (*) and *P* < 0.0001 (****). *n* represents the number of samples tested.

All 379 initially included samples were analyzed by the LAMP assay and the *PfvarATS/18S rRNA* multiplex qPCR. Both methods showed declining but persistent DNA detection (Fig. 3). At D28, 44% of samples were LAMP positive, 31% of samples were *18S rRNA* qPCR-positive, and 61% of samples were *PfvarATS* qPCR positive. No sample was positive for the *18S rRNA* target and negative by *PfvarATS*. Positivity rates for each time point are detailed in Table 1. Ct values increased over time, indicating decreasing DNA quantity.

**TABLE 1:** Positivity rates and mean Ct values of *Plasmodium* DNA detection during follow-up by LAMP and the *PfvarATS/18S rRNA* qPCR^*a*^

	LAMP assay	*PfvarATS* gDNA	Mean Ct ± SD	*18S rRNA* gDNA	Mean Ct ± SD
D0	100% (90/90)	100% (90/90)	17 ± 3	100% (90/90)	19 ± 3
D3 (1–4)	98% (83/85)	99% (84/85)	27 ± 4	96% (82/85)	29 ± 4
D7 (5–10)	83% (74/89)	94% (84/89)	30 ± 3	85% (76/89)	32 ± 3
D28 (21–35)	44% (44/100)	61% (61/100)	34 ± 2	31% (31/100)	35 ± 2

^*a*^ Ct, cycle threshold; SD, standard deviation.

These results demonstrate that *Plasmodium* DNA can persist for several weeks after treatment, reflecting a slow clearance of residual parasite genetic material.

Based on this screening, 61% of patients diagnosed with malaria remained *PfvarATS* DNA-positive through D28. However, only 58 patients had a complete follow-up (D0/D3 and D28), and thus, their corresponding samples were selected for further RNA analysis.

To investigate whether persistent molecular positivity was associated with the initial parasite burden, we correlated D28 molecular results with baseline parasitemia at D0. No measure of initial burden was significantly associated with the probability of remaining positive (all *P* ≥ 0.08). Unlike controlled human infection studies of untreated volunteers, initial parasite density did not predict clearance in this treated cohort, consistent with effective ACT-based therapy.

**Fig 4:**
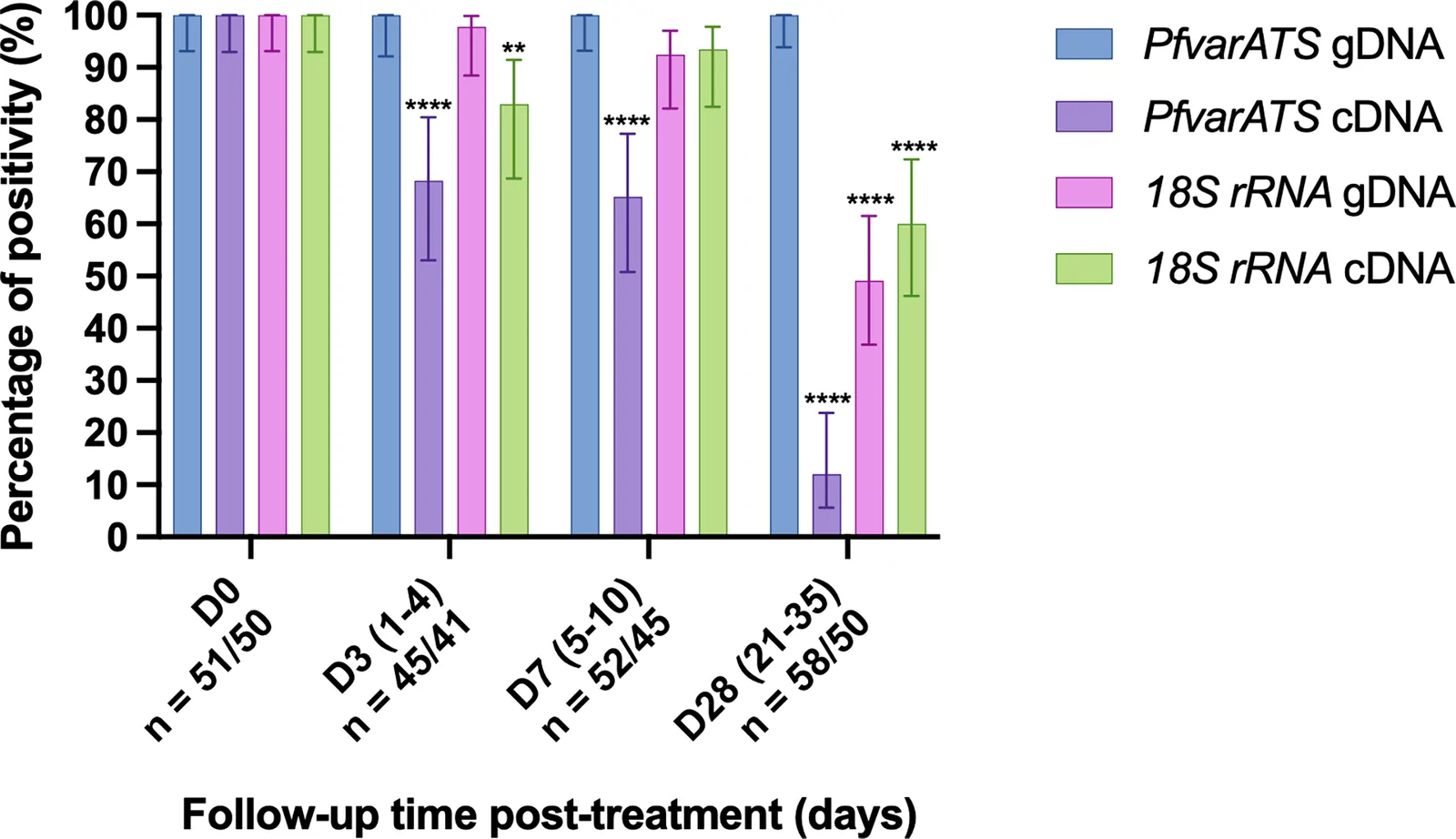
*PfvarATS/18S rRNA* qPCR positivity rates in *PfvarATS* gDNA-positive samples. qPCR assays targeting the *PfvarATS* and *18S rRNA* genes were performed on a subset of samples that remained positive by PfvarATS qPCR up to D28. For each follow-up time point, samples were classified as positive or negative for gDNA and cDNA, and the proportion of positive samples was calculated relative to the total number of samples analyzed at that time point. Results are expressed as percentages. Error bars represent 95% confidence intervals. Data were analyzed using GraphPad Prism v10.6.1. Statistical comparisons between follow-up time points and baseline (D0) for each marker were performed using Fisher’s exact test, *P* = 0.001 (**) and *P* < 0.0001 (****). *n* represents the number of samples tested (*n* = gDNA samples number/cDNA samples number).

Within this subset, we compared DNA (gDNA) and RNA (cDNA) detection for different stage markers to explore transcriptional activity (Fig. 4). Transcript detection (cDNA) declined faster than gDNA detection for *PfvarATS*, whereas the opposite was observed for the *18S rRNA* gene. In fact, while *PfvarATS* gDNA remained detectable until D28, its transcript detection, mainly reflecting ongoing asexual parasites’ transcriptional activity, dropped sharply from 100% (D0) to 12% (D28). In contrast, *18S rRNA* transcripts were more persistent, with 60% positivity at D28 versus 49% for its gDNA (Fig. 4 and Table 2).

**TABLE 2:** Positivity rates and mean Ct values of *Plasmodium* DNA and RNA detection during follow-up by the *PfvarATS/18S rRNA* qPCR^*a*^

	*PfvarATS* gDNA	Mean Ct ± SD	*PfvarATS* cDNA	Mean Ct ± SD	*18S rRNA* gDNA	Mean Ct ± SD	*18S rRNA* cDNA	Mean Ct ± SD
D0	100% (51/51)	17 ± 3	100% (50/50)^*b*^	25 ± 5	100% (51/51)	19 ± 3	100% (50/50)^*b*^	22 ± 6
D3 (1–4)	100% (45/45)	26 ± 4	68% (28/41)^*b*^	30 ± 5	98% (44/45)	28 ± 4	83% (34/41)^*b*^	26 ± 5
D7 (5–10)	100% (52/52)	29 ± 3	66% (30/45)^*b*^	33 ± 3	94% (49/52)	31 ± 3	95% (43/45)^*b*^	29 ± 4
D28 (>20)	100% (58/58)	34 ± 2	12% (6/50)^*b*^	35 ± 2	50% (29/58)	35 ± 2	60% (30/50)^*b*^	34 ± 3

^*a*^ Ct, cycle threshold; SD, standard deviation.

^*b*^ Some samples are missing due to reverse transcription inefficacy.

### Detection of gametocyte-specific markers

To evaluate *P. falciparum* gametocytogenesis in this cohort, transcripts of key sexual development markers (*PfAP2-G*, *Pfs16*, *PfCCP4*, and *PfMGET*) were analyzed by RT-qPCR on available samples.

*PfAP2-G* expression was highest at D0 (detected in 54% [19/35] of tested samples) and undetectable at D3 (Table 3).

**TABLE 3:** Positivity rates and mean Ct values of gametocyte transcript detection during follow-up^*a*^^,^^*c*^

	*PfCCP4* cDNA	Mean Ct ± SD	*PfMGET* cDNA	Mean Ct ± SD	PfAP2-G cDNA	Mean Ct ± SD	Pfs16 cDNA	Mean Ct ± SD
D0	42% (21/50)^*b*^	31 ± 6	30% (15/50)^*b*^	29 ± 6	54% (19/35)^*b*^	27 ± 4	100% (10/10)^*b*^	28 ± 6
D3 (1–4)	19% (8/41)^*b*^	31 ± 7	22% (9/41)^*b*^	30 ± 6	0 (0/14)	–	94% (15/16)^*b*^	33 ± 5
D7 (5–10)	33% (15/45)^*b*^	32 ± 6	31% (14/45)^*b*^	29 ± 5	–	–	67% (31/46)^*b*^	33 ± 4
D28 (>20)	10% (5/50)^*b*^	33 ± 5	12% (6/50)^*b*^	34 ± 2	–	–	–	–

^*a*^ Ct, cycle threshold; SD, standard deviation.

^*b*^ Some samples are missing due to reverse transcription inefficacy or RNA material availability.

^*c*^ "–" means the analysis was not performed for this time point or not applicable.

*Pfs16*, a pan-gametocyte marker, was detected in 100% (10/10) of tested samples at D0, 94% (15/16) at D3, and 67% (31/46) at D7 (Table 3), indicating robust expression that markedly exceeded that of mature gametocyte markers (*PfCCP4/PfMGET*) at the same time points. This suggests sexually committed ring stages and immature gametocytes predominated at D0 and D3/D7, respectively, as these forms express *Pfs16* but have not yet upregulated mature stage markers.

**Fig 5:**
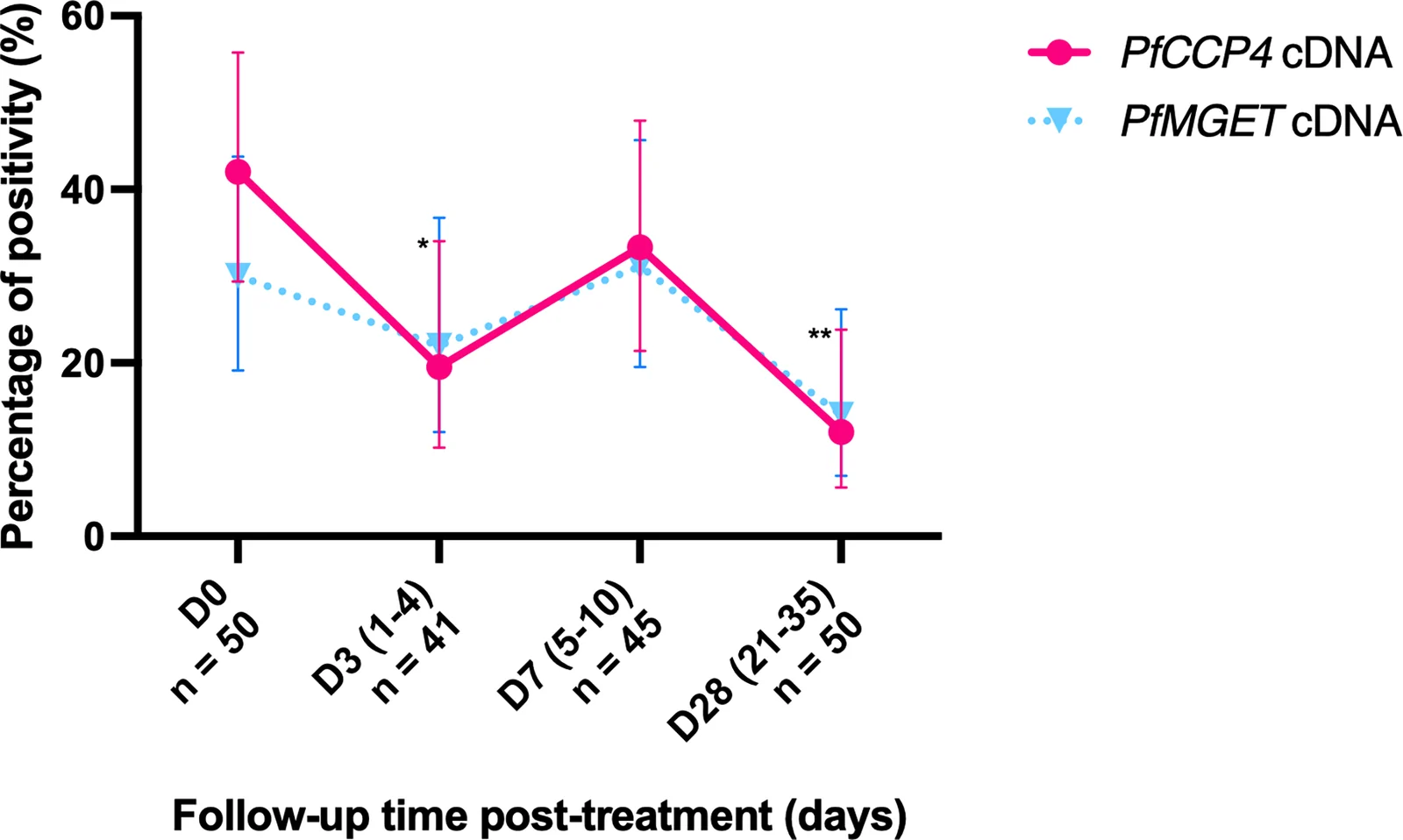
Detection of mature *Plasmodium falciparum* gametocyte markers by RT-qPCR during post-treatment follow-up. RT-qPCR targeting the *P. falciparum* gametocyte-specific markers *PfCCP4* and *PfMGET* was performed on the included positive samples to determine gametocyte carriage. The proportion of positive samples was calculated relative to the total number of samples analyzed at that time point. Results are expressed as percentages. Error bars represent 95% confidence intervals. Data were analyzed using GraphPad Prism v10.6.1. Statistical comparisons between follow-up time points and baseline (D0) for each marker were performed using Fisher’s exact test, *P* = 0.03 (*) and *P* = 0.002 (**). *n* represents the number of samples tested*.*

Mature gametocyte markers *PfCCP4* and *PfMGET* were detected from D0 through D28. Positivity rates were substantial at D0 (42% for PfCCP4 and 30% for *PfMGET*), decreased at D3 (19% and 22% respectively), and subsequently increased at D7 (33% and 31%), before persisting at low levels up to D28 (12% and 14%; Fig. 5 and Table 3).

Together, these data confirm that sexual commitment occurred at D0 (ongoing infection), leading to a population of committed trophozoites/immature gametocytes evident by D3 (high *Pfs16* and low *PfCCP4/PfMGET* expression), followed by maturation and gradual decline over time (increasing *PfCCP4/PfMGET* expression at D7 and a slow decrease until D28).

### Gametocyte carriage relative to treatment

Antimalarial drugs administered to the 58 patients included in this analysis were 39 DP, 12 AS, then DP, 4 AP, 2 Q, and 1 AL. The number of gametocyte-positive and gametocyte-negative samples at each time point was identified by the *PfCCP4/PfMGET* RT-qPCR.

Among the 45 patients evaluated at D3, 10 (22%) had detectable gametocytes. These patients were treated with DP (*n* = 4), AS then DP (*n* = 5), and AP (*n* = 1). Only two of the four patients receiving AP presented at the D3 checkup. No gametocyte carriage was observed for the AL and Q arms at this time point.

By D7, 15 out of 52 patients (29%) were gametocyte positive. This positivity was noted under DP (*n* = 9), AS then DP (*n* = 3), and AP (*n* = 3). No gametocyte carriage was observed in the sample from the patient treated with AL at D7.

At D28, 7 out of 58 patients (12%) remained gametocyte positive. These cases persisted under DP (*n* = 7). No gametocyte carriage was observed for AP, AS, then DP, AL, or Q at D28.

Due to the limited number of samples available for certain treatment groups, statistical analyses could not be reliably performed.

## DISCUSSION

The aim of this prospective study was to explore the influence of gametocytogenesis on the persistence of plasmodial DNA following treatment in a non-endemic setting. Our findings suggest that late qPCR positivity in treated patients predominantly reflects non-viable residual DNA rather than ongoing gametocyte circulation. Indeed, although 61% of patients remained qPCR positive at D28, only 12% had detectable transcripts of mature gametocytes, indicating a dissociation between molecular DNA persistence and true transmission potential. In this context, many studies have documented prolonged molecular assay positivity among returning travelers even after successful anti-malarial treatment (9, 19, 20, 29, 30). Some of these studies reported qPCR positivity up to 6–7 weeks post-treatment (9, 20). In some cohorts, more than half of patients remained qPCR-positive during late follow-up, highlighting the need for cautious interpretation of DNA-based molecular assays when assessing treatment efficacy (29). Our data align with previous reports of prolonged DNA detection in travelers, as we observed 44% and 61% positivity rates at D28 post-treatment by LAMP and qPCR methods, respectively, but extend them by simultaneously analyzing early (*PfAP2-G* and *Pfs16*) and mature (*PfCCP4*/*PfMGET*) gametocyte molecular markers. The multiplex RT-qPCR assay targeting the female and male transcripts *PfCCP4* and *PfMGET* is reportedly very sensitive, time-saving, and has already been used and validated in a field study to highlight the presence of gametocytes in asymptomatic malaria carriers (31). This multi-marker approach allows the distinction between persistent residual DNA, active gametocytogenesis, and viable stage V gametocytes, an essential differentiation that has been lacking in earlier work.

To distinguish residual non-viable parasite genetic material from viable parasites or sexual forms, an mRNA-based approach may be more appropriate than DNA-based detection. Gametocyte-specific mRNAs (such as *Pfs25*, *Pfs230*, *PfMGET*, *PfCCP4*...) are solely expressed at the mature sexual stages of *P. falciparum* and often at high copy numbers in active gametocytes (25, 32), which allows the specific identification of these forms.

Our results showed that while gDNA qPCR remained positive throughout the follow-up period, RT-qPCR positivity decreased more rapidly. *PfvarATS* transcript detection went from 68% at D3 to 12% at D28, while *18S rRNA* transcript positivity dropped to 83% at D3 and 60% by D28. *PfvarATS* mRNA predominantly reflects active transcription in asexual-stage parasites and therefore drops quickly once parasites are dead. The low levels of *PfvarATS* transcripts detected at D28 could nonetheless originate from viable gametocytes, which express this gene at substantially lower levels compared to asexual stages (33). In contrast, *18S rRNA* is present in all parasite stages, including gametocytes (34). The gametocyte dynamics observed, with a peak of mature gametocytes’ detection at D7 following early markers expression, match the expected timeline of gametocytogenesis (4). The detection of mature gametocyte transcripts at D0 likely reflects both true carriage from previous replication cycles, since 0.2%–1% of asexual parasites undergo sexual commitment at every replication cycle (35), and cross-detection from high asexual parasitemia as reported by Meerstein-Kessel et al. (25). That was the case in our cohort, as 96% of gametocyte-positive D0 samples had a parasitemia higher than 1,000 parasites/µL, as determined by microscopy. Furthermore, the predominance of *Pfs16* transcripts at D0 and D3 indicates that most gametocytes present early after treatment are likely committed forms or immature stages (high *Pfs16* and low *PfCCP4/PfMGET* expression).

The notable persistence of *18S rRNA* transcripts compared to gametocyte-specific transcripts could have many explanations. For one, the different targets’ sensitivity, as demonstrated by our sensitivity assays. Therefore, RNA from persisting gametocytes not detected by the *PfCCP4/PfMGET* RT-qPCR can be observed at later time points. Indeed, gametocyte-specific genes are expressed at lower copy numbers compared to *18S rRNA*, and their transcripts decay rapidly following the disappearance of mature gametocytes, so negative results due to the assay’s lower sensitivity are possible. In this context, a Tanzanian community survey demonstrated that *18S rRNA*-positive samples were over sixfold more common than those with detectable *Pfs25* transcripts by RT-qPCR, highlighting the higher abundance and detectability of ribosomal transcripts compared to mature gametocyte-specific transcripts (36). Moreover, ribosomal RNA is very abundant in eukaryotes and is structurally protected within ribonucleoprotein complexes, slowing its degradation (37). Consequently, *18S rRNA* could be detected for an extended period. As a matter of fact, several cohort studies have documented persistent *18S rRNA* signals by RT-qPCR for days to weeks post-treatment (9, 38). Interestingly, one study combining viability dyes like propidium monoazide with RT-qPCR has even demonstrated that a significant portion of PCR-detectable parasite RNA can originate from non-viable organisms (39). Therefore, we hypothesize that the substantial persistence of *18S rRNA* transcripts at D28 compared to the other markers does not exclusively indicate the presence of viable gametocytes; the observed results likely represent a combination of low-level transcription from any remaining gametocytes and a more substantial contribution from conserved residual RNA. It would be interesting to integrate an asexual stage marker such as Skeleton binding protein 1 (SBP1), one of the most abundant proteins expressed during the asexual intraerythrocytic stages, particularly trophozoites and schizonts (40), in future RT-qPCR studies to help determine whether the detected RNA signal originates from sexual or asexual parasite stages.

Our results suggest that treatment could influence the outcome of gametocytes, as active gametocytogenesis was evident in early follow-up (high *PfAP2-G*/*Pfs16* transcripts), yet mature gametocytes were scarce by D28. This dissociation suggests that some antimalarial treatments, although unable to completely suppress sexual commitment, could be effective in restricting the development or circulation of mature infectious gametocytes. Although exploratory observations suggested differences in gametocyte carriage according to antimalarial regimen, the number of patients receiving treatments other than DP was too limited to allow meaningful comparisons. Therefore, treatment-specific findings should be interpreted with caution and considered hypothesis-generating only. Larger prospective studies specifically designed to evaluate the impact of treatment on gametocyte dynamics are still required to address this question.

This study has a few limitations that should be considered when interpreting our data. First, gametocyte detection relied on RT-qPCR targeting parasite mRNA, which is inherently fragile and very sensitive to sample handling and storage conditions. Other factors could also have an impact, such as the low density of circulating gametocytes, the analytical sensitivity of the different assays, and the efficiency of reverse transcription. Although the assay sensitivity was estimated using serial dilutions tested in triplicate, a more comprehensive analytical validation, including a larger number of replicates and statistical determination of the LoD, would strengthen the characterization of assay performance. Therefore, an underestimation of true gametocyte carriage could not be excluded. In addition, RNA sample availability varied, resulting in smaller subsets for some transcript analyses, particularly *PfAP2-G* and *Pfs16*. These markers were investigated at a later stage of the study, after the analysis of the mature gametocyte markers and the other PCR assays had been completed; thus, the remaining sample material was insufficient to test the entire cohort. Nevertheless, the observed transcriptional patterns remained biologically consistent with the expected sequence of sexual commitment, immature gametocyte development, and subsequent maturation. Finally, we focused on gametocyte transcript detection and not quantification, which could have provided more insight into gametocytogenesis kinetics under treatment.

In conclusion, persistent *P. falciparum* DNA detection up to 28 days post-treatment in a non-endemic setting is most consistent with residual parasite genetic material and, in a minority of cases, detectable circulating mature gametocytes. These findings are critical for molecular biology test interpretation in non-endemic areas. As molecular tests (including point-of-care molecular tests such as LAMP) are increasingly popular, clinicians and microbiologists must remain cautious in interpreting positive PCR in the context of therapeutic efficacy or previous treatment. Interpretation must integrate clinical status, microscopy, and timing. Incorporating gametocyte-specific markers could help differentiate residual DNA from potential transmission reservoirs, though their low density poses detection challenges.

Future work should aim to understand the decay kinetics of nucleic acids in the organism and evaluate whether integrating gametocyte-specific markers into the post-treatment follow-up process could improve the interpretation of molecular results.

## Data Availability

This study is a prospective clinical cohort with anonymized patient data (clinical and epidemiological information, microscopy, and molecular tests results). All data supporting the findings of this study are included within the article and its supplemental material.

## SUPPLEMENTAL MATERIAL

File S1 spectrum.02312-26-s0001.xlsx Patient data.

Fig. S1 spectrum.02312-26-s0002.tiff Assay specificity analysis.

Tables S1 and S2 spectrum.02312-26-s0003.docx qPCR assays reaction details.

ASM does not own the copyrights to Supplemental Material that may be linked to, or accessed through, an article. The authors have granted ASM a non-exclusive, world-wide license to publish the Supplemental Material files. Please contact the corresponding author directly for reuse.
