# Supplementary material for: Persistence of Plasmodium falciparum DNA detection after antimalarial treatment and the implication of gametocytes: a prospective cohort study in a non-endemic setting
Source: Microbiol Spectr. 2026 Sep 2;14(10):e02312-26. doi: 10.1128/spectrum.02312-26 (PMC13689117; doi:10.1128/spectrum.02312-26)
Supplement: Supplementary file 1 [file spectrum.02312-26-s0003.docx]

**Table S1: Oligonucleotides sequences of the different assays in this study**

| **Oligo name** | **Oligo sequence** | **Oligo modification [5’-3’]** | **Final concentration**  **[µM]** | **Adapted from** |
| --- | --- | --- | --- | --- |
| **1) *PfvarATS/18S rRNA* assay** |  |  |  |  |
| Pspp18S fwd | GCT CTT TCT TGA TTT CTT GGA TG | - | 0,4 | Schindler et al, 2019 (23) |
| Pspp18S rev | AGC AGG TTA AGA TCT CG TTC G | - | 0,4 | Schindler et al, 2019 (23) |
| Pspp18S probe | ATG GCC GTT TTT AGT TCG TG | Cy5-BHQ1 | 0,2 | Schindler et al, 2019 (23) |
| HsRNaseP fwd | AGA TTT GGA CCT GCG AGC G | - | 0,2 | Schindler et al, 2019 (23) |
| HsRNaseP rev | GAG CGG CTG TCT CCA CAA GT | - | 0,2 | Schindler et al, 2019 (23) |
| HsRNaseP probe | TTC TGA CCT GAA GGC TCT GCG CG | VIC-BHQ1 | 0,1 | Schindler et al, 2019 (23) |
| PfvarATS fwd | CCC ATA CAC AAC CAA YTG GA | - | 0,4 | Schindler et al, 2019 (23) |
| PfvarATS rev | TTC GCA CAT ATC TCT ATG TCT ATC T | - | 0,4 | Schindler et al, 2019 (23) |
| vPfvarATS probe | TRT TCC ATA AAT GGT | FAM-MGB | 0,25 | Schindler et al, 2019 (23) |
| **2) *PfCCP4/PfMGET* assay** |  |  |  |  |
| PfCCP4 fwd | CAC ATG AAT ATG AGA ATA AAA TTG | - | 0,9 | Meerstein‐Kessel et al, 2018 (24) |
| PfCCP4 rev | TAG GCG AAC ATG TGG AAA G | - | 0,9 | Meerstein‐Kessel et al, 2018 (24) |
| PfCCP4 probe | AGC AAC AAC GGT ATG TGC CTT AAA ACG | VIC-BHQ1 | 0,2 | Meerstein‐Kessel et al, 2018 (24) |
| PfMGET fwd | CGG TCC AAA TAT AAA ATC CTG | - | 0,225 | Meerstein‐Kessel et al, 2018 (24) |
| PfMGET rev | TGT GTA ACG TAT GAT TCA TTT TC | - | 0,225 | Meerstein‐Kessel et al, 2018 (24) |
| PfMGET probe | CAG CTC CAG CAT TAA AAA CAC | FAM-BHQ1 | 0,2 | Meerstein‐Kessel et al, 2018 (24) |
| **3) *PfAP2-G* and *PfS16* assays** |  |  |  |  |
| PfAP2-G fwd | TGT TCT GTA CCT GTA GTT GTG GA | - | 0,4 | - |
| PfAP2-G rev | CAT CTT CGT CAT CAT CAT TTC CA | - | 0,4 | - |
| PfAP2-G probe | CCG GAA GTA AAC AAA GTG TGA | HEX-BHQ1 | 1,5 | - |
| Pfs16 fwd | AAT CAA GGT CTA TCT CAA GG | - | 0,4 | - |
| Pfs16 rev | TCA TCT CCT TCG TCT CCT TCA | - | 0,4 | - |
| Pfs16 probe | ACG CAG ATG ATG ATA AAG GAG A | FAM-BHQ1 | 2 | - |

**Table S2 : Assays reaction and cycling conditions**

|  | *PfvarATS/18S rRNA* assay | *PfCCP4/PfMGET* assay | *PfAP2-G* assay | *PfS16* assay |
| --- | --- | --- | --- | --- |
| qPCR reaction conditions |  |  |  |  |
| Total volume | 20 µL | 20 µL | 20 µL | 20 µL |
| DNA volume | 6 µL | 5 µL | 5 µL | 5 µL |
| 2X Luna® Universal probe qPCR Master Mix (NewEngland Biolabs) | 1X | 1X | 1X | 1X |
| qPCR cycling conditions |  |  |  |  |
| Polymerase activation | 1 min − 95°C | 1 min − 95°C | 1 min − 95°C | 1 min − 95°C |
| Denaturation | 15 s − 95°C | 10 s − 95°C | 15 s − 95°C | 15 s − 95°C |
| Annealing & Elongation | 45 s − 55°C | 1 min − 60°C | 30 s − 58°C | 30 s − 58°C |
| Number of cycles | 45 | 45 | 45 | 45 |
